# Methodological biases in observational hospital studies of COVID-19 treatment effectiveness: pitfalls and potential

**DOI:** 10.3389/fmed.2024.1362192

**Published:** 2024-03-21

**Authors:** Oksana Martinuka, Derek Hazard, Hamid Reza Marateb, Marjan Mansourian, Miguel Ángel Mañanas, Sergio Romero, Manuel Rubio-Rivas, Martin Wolkewitz

**Affiliations:** ^1^Institute of Medical Biometry and Statistics, Faculty of Medicine and Medical Center - University of Freiburg, Freiburg, Germany; ^2^Biomedical Engineering Research Center (CREB), Automatic Control Department (ESAII), Universitat Politècnica de Catalunya-Barcelona Tech (UPC), Barcelona, Spain; ^3^Department of Artificial Intelligence, Smart University of Medical Sciences, Tehran, Iran; ^4^Department of Epidemiology and Biostatistics, School of Health, Isfahan University of Medical Sciences, Isfahan, Iran; ^5^CIBER de Bioingeniería, Biomateriales y Nanomedicina (CIBER-BBN), Madrid, Spain; ^6^Department of Internal Medicine, Bellvitge University Hospital, Hospitalet de Llobregat, Barcelona, Spain

**Keywords:** competing risks, confounding, COVID-19, emulated trial, immortal-time bias, methodological bias, treatment effectiveness

## Abstract

**Introduction:**

This study aims to discuss and assess the impact of three prevalent methodological biases: competing risks, immortal-time bias, and confounding bias in real-world observational studies evaluating treatment effectiveness. We use a demonstrative observational data example of COVID-19 patients to assess the impact of these biases and propose potential solutions.

**Methods:**

We describe competing risks, immortal-time bias, and time-fixed confounding bias by evaluating treatment effectiveness in hospitalized patients with COVID-19. For our demonstrative analysis, we use observational data from the registry of patients with COVID-19 who were admitted to the Bellvitge University Hospital in Spain from March 2020 to February 2021 and met our predefined inclusion criteria. We compare estimates of a single-dose, time-dependent treatment with the standard of care. We analyze the treatment effectiveness using common statistical approaches, either by ignoring or only partially accounting for the methodological biases. To address these challenges, we emulate a target trial through the clone-censor-weight approach.

**Results:**

Overlooking competing risk bias and employing the naïve Kaplan-Meier estimator led to increased in-hospital death probabilities in patients with COVID-19. Specifically, in the treatment effectiveness analysis, the Kaplan-Meier estimator resulted in an in-hospital mortality of 45.6% for treated patients and 59.0% for untreated patients. In contrast, employing an emulated trial framework with the weighted Aalen-Johansen estimator, we observed that in-hospital death probabilities were reduced to 27.9% in the “X”-treated arm and 40.1% in the non-“X”-treated arm. Immortal-time bias led to an underestimated hazard ratio of treatment.

**Conclusion:**

Overlooking competing risks, immortal-time bias, and confounding bias leads to shifted estimates of treatment effects. Applying the naïve Kaplan-Meier method resulted in the most biased results and overestimated probabilities for the primary outcome in analyses of hospital data from COVID-19 patients. This overestimation could mislead clinical decision-making. Both immortal-time bias and confounding bias must be addressed in assessments of treatment effectiveness. The trial emulation framework offers a potential solution to address all three methodological biases.

## Introduction

During the coronavirus disease 2019 (COVID-19) pandemic, routinely collected observational data has become crucial for comparative treatment effectiveness research and for identifying potential therapeutic options (1, 2). Real-world observational data was increasingly used during the pandemic’s first waves when results from randomized clinical trials were either unavailable or used to complement trial findings. Observational studies can yield biased results when they are not appropriately designed and analyzed because of their type of data and potential methodological challenges (1, 3–5). While the methodological limitations of observational data have been extensively discussed, a review of early observational studies on the effectiveness of repurposed or novel treatments for COVID-19 patients indicated that fundamental methodological biases such as competing risks, immortal-time bias, and confounding bias, either alone or in combination, were still often overlooked (2). Failure to address these methodological biases can result in skewed estimates of treatment effects and, consequently, incorrect conclusions (2, 5).

A competing risk is an event that precludes the observation of the primary event of interest (6, 7). In COVID-19 studies, when in-hospital mortality is the primary outcome, discharge becomes a competing event because it hinders the observation of death in hospital (8). Conventional survival analysis techniques, such as the naïve Kaplan-Meier estimator, treat competing events as right-censored observations. This approach assumes that censored individuals will have the same probability of experiencing the event of interest as those who remain in the risk set, leading to a positive event probability instead of zero probability after the occurrence of a competing event (7, 9–11). For comprehensive mathematical proofs, we refer to the studies conducted by Zhang (11) and Coemans et al. (10). In the context of COVID-19 and analyzing in-hospital death, this assumption would imply that discharged patients have a similar risk of death as those still hospitalized, which is not clinically meaningful (7, 12). Hence, the independent censoring assumption is violated for hospital discharge because discharged patients are usually in better health conditions than those still hospitalized (13). In the presence of competing events, the naïve Kaplan-Meier method can lead to biased estimates and erroneous conclusions (13). Notably, the issue of competing risks can arise in analyzing time-to-event survival data in randomized clinical trials, observational studies, and target trial emulations (6).

Observational studies often evaluate the effectiveness of time-dependent treatments, meaning patients may initiate treatment at different times after their study entry (14). Immortal time occurs when there is a delay between cohort entry and treatment initiation, during which patients are precluded from experiencing the outcome. Misclassifying or excluding this pre-treatment period can introduce immortal-time bias, thereby biasing the estimated treatment effects (15–17). Previous studies have demonstrated that the most severe form of immortal-time bias occurs when studies incorrectly include immortal time, assuming that treated patients are at risk from the baseline. This is in contrast to methods designed to mitigate this bias, such as landmark analysis, the exposure density sampling method, and the time-dependent Cox model with time-varying treatment status (18–20). When immortal time is mistakenly included, it leads to an artificially reduced observed event rate for the treatment group and an artificially inflated event rate for the control group (14, 21). As a result, the hazard ratio (HR) for comparing the treatment vs. the control group may be underestimated (20). For negative outcomes like death, such underestimation misleadingly suggests a greater treatment effectiveness. In contrast, for positive outcomes like discharge, the underestimation of the treatment effect can make the treatment appear less effective. For a comprehensive review of the mathematical proofs, we refer to the studies conducted by Suissa (20), Beyersmann et al. (22), and the simulation study by Wang et al. (19).

Confounding represents another well-known and significant challenge in observational studies, arising from an unequal distribution of patient characteristics between treatment and control groups, which affect both treatment decision and outcome (23, 24). Therefore, simply comparing outcomes between the treatment and control groups without any adjustment can lead to biased estimates of treatment effects (25, 26). In causal analyses, common approaches to adjust for baseline characteristics include inverse probability weighting, standardization, and stratification-based adjustment methods such as stratification and matching methods (27, 28).

Throughout the COVID-19 pandemic, the target trial emulation framework was widely used to assess the effectiveness of treatments and vaccines using real-world data, particularly in the pandemic’s early stages (29–32). This framework applies the principles of randomized clinical trials to emulate a hypothetical trial using observational data, thereby answering specific causal questions (24, 33). It has become crucial to explore treatment effects and address common methodological biases (34). While previous research has demonstrated that target trial emulation can handle both immortal-time bias and confounding bias, our study further confirms the importance of considering competing risks within observational data (19, 34).

The aim of this study is 3-fold: (i) to provide an overview of the three most common methodological biases in observational hospital data; (ii) to evaluate the impact of each bias using a typical example of observational hospital data and applying various analytical methodologies; and (iii) to describe the target trial emulation framework that addresses these potential methodological challenges. For illustrative purposes, we analyzed observational hospital data from patients with COVID-19. This article provides an explanation of the potential methodological pitfalls in a descriptive manner and proposes alternative strategies for mitigating these challenges.

## Methods

The Methods section is organized as follows: we introduce challenges associated with competing risks through a typical example of observational hospital data of COVID-19 patients and conduct a time-to-event analysis without accounting for the patient’s treatment status. We then describe a cohort of patients used for our demonstrative analyses and introduce the concept of target trial emulation. Next, we discuss immortal-time and confounding biases, outline standard analysis methods prone to bias, and explain how these challenges can be mitigated within the emulated trial framework. We define the five models used for comparison to determine the impact of immortal-time bias and confounding bias. We emphasize that all analyses conducted, including the emulated trial, were demonstrative, and an assessment of clinical treatment effects was beyond the scope of this study.

### Motivating example: competing risks in a COVID-19 hospital setting

To illustrate the concept of competing risks in time-to-event analysis of hospital data, we conducted an analysis using longitudinal patient-level data from a cohort of COVID-19 patients (*n* = 478) hospitalized at the Bellvitge University Hospital in Barcelona, Spain, from March 2020 to February 2021. These patients experienced various endpoints, including in-hospital death, discharge home, or transfer to another healthcare facilities. In this analysis, we defined in-hospital death as the primary outcome of interest and estimated the cumulative probabilities without considering the patient’s treatment status. Information on patient survival status beyond the follow-up period was not available.

In the naïve analysis, we calculated the cumulative probabilities using the one minus Kaplan-Meier estimator. We compared these results with those from the Fine-Gray analysis approach, which accounts for competing events like hospital discharge by keeping patients in the risk set until the end of follow-up. The Fine-Gray model is a direct model for cumulative incidence functions in the presence of competing risks (35). We conducted two Fine-Gray analyses. In the first analysis, we treated patients discharged home as a competing event and considered patients transferred to other facilities as censored observations, thus implementing the Fine-Gray model with two events. In the second analysis, we distinguished between reasons for hospital discharge, categorizing discharge to home and transfer to another healthcare facility as separate competing events. This approach allowed us to maintain both outcomes in the risk set, corresponding to the Fine-Gray model with three events.

**Figure 1:**
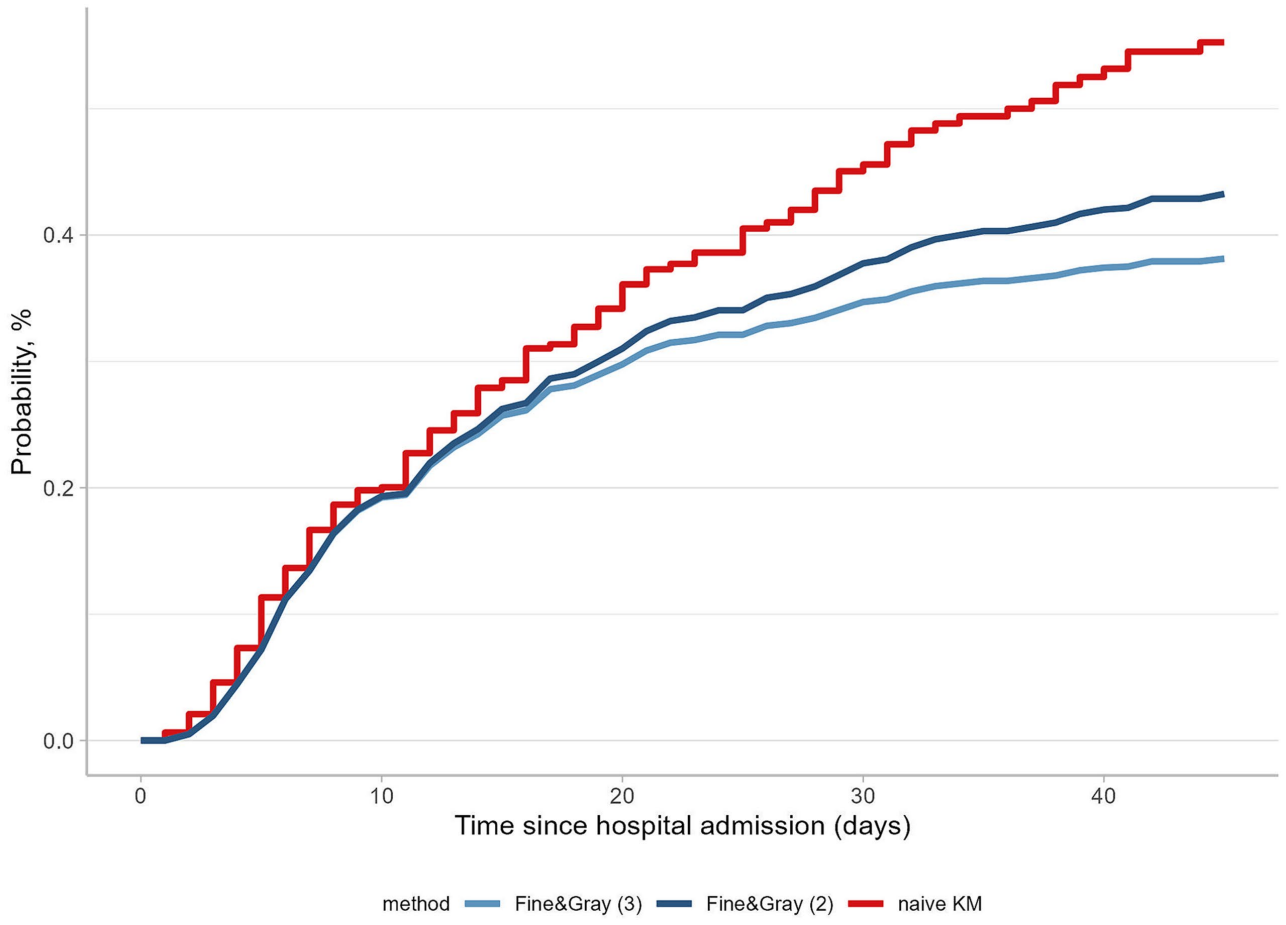
Probabilities of in-hospital death with and without accounting for competing events. Probabilities of in-hospital death are calculated taking different analytical approaches: the Fine-Gray (3) model, considering three outcomes; the Fine-Gray (2) model, considering two outcomes, and the naïve analysis using the one minus the Kaplan-Meier estimator.

Using the naïve Kaplan-Meier estimator resulted in an overestimated in-hospital death probability of 55.3% (Figure 1). By recognizing discharge home as the only competing event and by censoring transferred patients, the probability of in-hospital death dropped to 43.3% (Figure 1). Finally, by considering both reasons for hospital discharge, the in-hospital death probability substantially decreased to 38.1% (Figure 1). These findings underscore the importance of recognizing and addressing competing risks in the hospital data and have also motivated us to explore the future extensions of emulated target trial methodologies.

### Illustrative study population: patients with COVID-19

For this case study, we analyzed longitudinal data from hospitalized patients with COVID-19 as described above. A total of 478 patients with moderate-to-severe COVID-19 were included. Inclusion criteria for these patients were based on the Horowitz index, a ratio of the partial pressure of oxygen to the fraction of inspired oxygen (PaO_2_/FiO_2_) of less than 300 mmHg measured at hospital admission and the presence of at least one inflammation-related high-risk factor: C-reactive protein (>102 mg/L), lactate dehydrogenase (>394 U/L), D-dimer (>1,580 ng/mL), total lymphocyte count (<760 × 10^6^/L), and ferritin (>1,360 mcg/L) at the time of admission. The high-risk categories were determined following the criteria and classification established by Rubio-Rivas et al. (36). For all patients, severe acute respiratory syndrome coronavirus 2 (SARS-CoV-2) infection was confirmed via PCR testing. The study follow-up period was 45 days post-hospital admission. Patients with no outcomes who were still alive at the end of this period were administratively censored (*n* = 59, 12.3%).

### Trial emulation: study question and protocol components

To emulate a target trial, we defined our clinical aim as follows: *to evaluate the effectiveness of treatment “X” compared to the standard-of-care, which does not involve the administration of treatment “X,” on the risk of in-hospital death while acknowledging its effects on hospital discharge outcomes in COVID-19 patients.* This question of interest could be subdivided into three distinct components: *assessing the impact of treatment on (i) in-hospital death, (ii) discharge alive home, and (iii) transfer to another healthcare facility*. We designed a hypothetical study protocol, specifying its components including eligibility criteria, treatment strategies and assignment, start and end of follow-up, endpoints, and causal contrast (Supplementary material 1).

### Immortal-time bias

In studies evaluating time-varying or time-dependent treatments addressing immortal-time bias is crucial, for which several options are available. Two commonly used approaches that can lead to severe immortal-time bias and result in flawed estimates of treatment effect: (i) including person-time and classifying patients as treated from time zero, even if they receive treatment later during follow-up, and (ii) excluding person-time, which is the time from baseline to treatment initiation for the exposure group (16, 19, 37, 38). The landmark analysis is a design-based method involving setting fixed time as the landmark time and classifying patients according to their treatment status at the landmark (17). Patients are then followed from the landmark time regardless of subsequent changes in their treatment status (17, 37). However, this approach has two principal limitations: (i) the choice of the landmark time and (ii) the exclusion of patients who had an outcome before the landmark time from the analysis (15, 28). To overcome these drawbacks, considering multiple landmarks and a pooled analysis via a supermodel is recommended (39). In the exposure density sampling method, unexposed patients are matched to exposed patients with respect to a time-dependent exposure. Specifically, for each exposed patient, one or more unexposed patients who have survived for a duration equivalent to that of the exposed patient are selected (40). This approach allows for the possibility that an unexposed patient may change their exposure status after matching. A simulation study demonstrated that the exposure density sampling method fully addressed immortal-time bias (40), in contrast to the simpler method of prescription time-distribution matching (18, 41). Another common approach to account for immortal person-time is to use a time-dependent model (16, 18, 19). It involves modeling time-varying treatment status and includes it as a time-dependent covariate in a proportional hazards or another regression model (19). This approach enables the classification of patients as “treated” or “untreated” on each follow-up day, allowing for the reclassification of patients from “untreated” to “treated” status upon the treatment’s initiation. Alternatively, clone-censor-weight and the sequential trial approaches allow for the incorporation of time-dependent treatment status through duplication or a nested design, and can be applied within the framework of trial emulation. The cloning approach creates two exact copies of each patient, assigning one clone to the treatment and the other to the control arm. Subsequently, a clone in each arm is censored when the actual treatment received deviates from the treatment strategy of the arm to which it was initially assigned (34). This usually requires defining a clinically meaningful grace period (33, 34). In the sequential trial approach, a sequence of multiple nested trials with all potential time zeros is modeled (37). Each method has its own assumptions and limitations, which should be considered when interpreting study results. Our study focuses on three approaches: analysis that includes immortal time, modeling time-varying treatment status and using time-dependent Cox regression model, and the clone-censor-weight approach.

In our illustrative observational data example, time zero, or the baseline, was defined as hospital admission, with the possibility of administering treatment at a later follow-up time. Consequently, patients’ treatment status depended on their presence in the risk set until a specific time. To evaluate the impact of included immortal time, we initially conducted a naïve analysis, mistakenly categorizing patients who received treatment during follow-up as having been treated since hospital admission (Model 1, Table 1 in the Results). In this instance, the time period between hospital admission and “X” treatment administration is immortal, as patients must be outcome-free to be categorized as treated (16). We also performed a time-dependent Cox regression analysis by modeling a time-varying treatment status using start-stop notation (Models 2–4). We used the clone-censor-weight approach for the target trial emulation, defining the grace period as treatment administration within 2 days of hospital admission (Model 5), as elaborated in Supplementary material 2. The length of this period was based on clinical relevance. We defined two treatment strategies: (1) administration of “X” treatment during the first 2 days of hospital admission, referred to as the “X”-treated arm, and (2) no administration of “X” treatment during the first 2 days, referred to the non-“X”-treated arm. Patients who experienced outcome events within 2 days were included in both treatment arms, avoiding immortal-time bias (34).

**Table 1:** Overview of statistical methods and results while addressing vs. neglecting immortal time and confounding biases.

Model	Approach	Statistical analysis method for outcome models	Hazard ratio (HR, [95% CI])			Immortal-time bias		Baseline confounding bias	
			In-hospital death	Discharge home	Transfer	Occurrence	Description	Occurrence	Description
1	Conventional	Univariable Cox regression model with treatment status incorrectly assigned at baseline	0.66 [0.47–0.93]	0.84 [0.59–1.21]	1.30 [0.86–1.94]	Yes	Ever-treated patients misclassified as treated from admission; never-treated as untreated	Yes	Baseline covariates not included in regression model
2	Conventional	Univariable, time-dependent Cox regression model with time-varying treatment status	0.79 [0.59–1.06]	0.91 [0.66–1.25]	1.38 [0.96–1.97]	No	Treated patients time classified to “untreated / “treated” periods using start-stop notation; pre-treatment time classified as “untreated”	Yes	
3	Conventional	Multivariable, time-dependent Cox regression model with baseline covariates and time-varying treatment status	0.76 [0.58–1.00]	0.92 [0.68–1.24]	1.41 [1.01–1.99]	No		No	Baseline covariates included in regression model
4	Inverse probability treatment weighting	Weighted, time-dependent Cox regression model with weights as a covariate and time-varying treatment status	0.76 [0.52–1.08]	0.98 [0.67–1.42]	1.50 [1.00–2.24]	No		No	Baseline covariates included in inverse probability treatment weights via propensity scores
5	Target trial emulation with clone-censor-weight approach	Weighted cause-specific Cox regression with censoring weights as a covariate and treatment arm	0.68 [0.46–1.02]	1.22 [0.82–1.81]	1.26 [0.77–2.07]	No	Two clones: one in ‘X’-treated arm and one in non-‘X’-treated arm	No	Cloning results in balanced covariates between two arms at baseline, inverse probability censoring weights applied to correct for selection bias

HR, Hazard ratio; CI, Confidence interval.

### Confounding bias

After identifying and collecting all important variables—potential confounders, several statistical approaches can be considered to mitigate confounding bias. We included the following patient baseline covariates in our study: age, sex, Charlson Comorbidity Index, levels of C-reactive protein, lactate dehydrogenase, D-dimer, total lymphocyte count, ferritin, and calendar time of hospital admission, categorized according to the pandemic waves. After examining the distribution of inflammatory variables, we applied the log and square root transformations to these variables to reduce the influence of extreme values. We assumed all these measured covariates were sufficient for controlling baseline confounding.

We first performed a univariable analysis without adjusting for baseline covariates to demonstrate the impact of ignoring time-fixed confounding (Models 1 and 2, Table 1). We then included the baseline covariates into a Cox regression model and performed multivariable analysis (Model 3). We also employed an inverse probability of treatment weighting model based on propensity scores to balance baseline covariates in the treatment and control groups (Model 4) (42). To balance the patient’s characteristics and prognostic covariates between treated and untreated groups, we re-weighted the outcome variables of these patients by the inverse probability of the treatment received (28, 43). As a result, we re-weighted the patients and created a pseudo-population free of confounding (42). We used the ipw package and calculated robust standard errors (44). In emulated trial analysis, we applied the clone-censor-weight approach (Model 5). Cloning patients into two arms ensured that the two arms were balanced regarding baseline covariates, addressing time-fixed confounding bias (34, 45). Additionally, to correct for selection bias resulting from artificial censoring, we estimated inverse probability of censoring weights (34). We applied the code as presented by Maringe et al. (34) for the target trial emulation analysis. Standardized differences were assessed before and after applying inverse probability of censoring weighting (Supplementary material 3). For this model, nonparametric bootstrap was used to compute 95% normal-based confidence intervals (CI) with 500 bootstrap replications. Multiple imputations were performed to replace missing values for inflammatory covariates measured at baseline. All analysis steps were applied to the five copies of the imputed datasets. Further details on the multiple imputation analysis are found in Supplementary material 4. All statistical analyses were performed in RStudio (2022.07.1) software (46).

## Results

### Patients characteristics

Overall, among the 478 patients with COVID-19 included in our initial data analysis, 183 (38.3%) experienced in-hospital death, 237 (49.6%) were discharged from the hospital, and 59 (12.3%) were administratively censored at the end of the 45-day follow-up period. Among the 237 discharged patients, 139 (58.6%) were discharged to their homes, while 98 (41.4%) were transferred to other healthcare facilities. In total, 143 (29.9%) patients were treated with “X” treatment at any time during the follow-up period. In the emulated trial analysis, 73 (15.3%) patients received the “X” treatment within 2 days. Among those who received the treatment, 20 died, 26 were discharged home, and 19 were transferred to other healthcare facilities. The cohort’s characteristics are detailed in Supplementary material 5.

### Assessing the impact of treatment on in-hospital death rates

**Figure 2:**
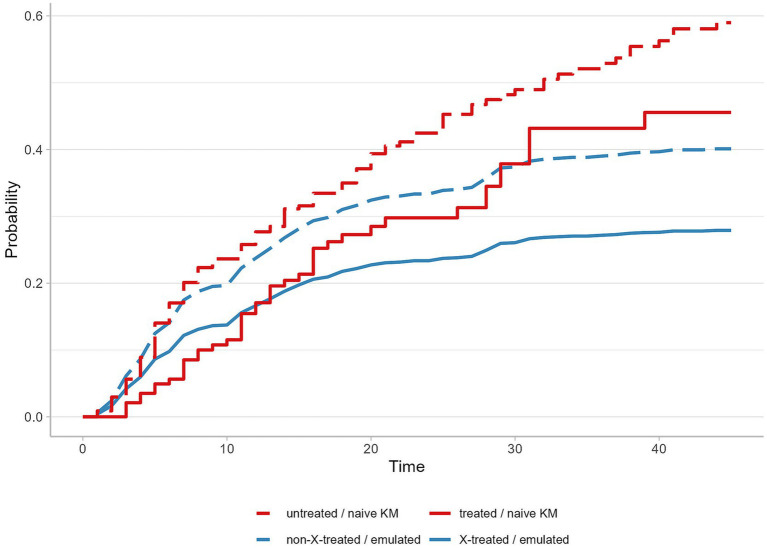
Cumulative in-hospital death probabilities by treatment group, comparing results from the naïve Kaplan-Meier estimator applied to initial data with the weighted Aalen-Johansen in emulated trial. Abbreviations: KM, the Kaplan-Meier estimator; Emulated, emulated target trial analysis using the weighted Aalen-Johansen estimator.

We calculated the cumulative incidence probabilities for in-hospital death by ignoring or accounting for competing events. Probabilities were derived using the conventional, naïve Kaplan-Meier estimator applied to the crude dataset, which was susceptible to all three biases. These results were compared to probabilities estimated from the weighted version of the Aalen-Johansen estimator used in the emulated analysis with the clone-censor-weight approach. The cumulative probabilities of in-hospital death using the naïve Kaplan-Meier estimator were 45.6% for the treated and 59.0% for the untreated group at the end of the 45-day follow-up period. In contrast, the Aalen-Johansen estimator revealed cumulative probabilities of 27.9% for the “X”-treated arm and 40.1% for the non-“X”-treated arm (Figure 2).

### Estimating treatment effects with and without addressing immortal time and confounding biases

We estimated the treatment effect while either ignoring or acknowledging immortal time and confounding biases, taking different approaches for three endpoints (Table 1). Model 1, which ignored both immortal-time and confounding biases, showed a significant decrease in in-hospital death with a resulting HR of 0.66 (95% CI, 0.47–0.93). In Model 1, the estimated effect for competing events was 0.84 (95% CI, 0.59–1.21) for discharge home and 1.30 (95% CI, 0.86–1.94) for transfer to another healthcare facility. By accounting for a delay in treatment administration time through modeling a time-varying treatment status in Model 2, the HRs increased for all outcomes: 0.79 (95% CI, 0.59–1.06) for in-hospital death, 0.91 (95% CI, 0.66–1.25) for discharge home, and 1.38 (95% CI, 0.96–1.97) for transfer. In addition, after adjusting for baseline covariates in Models 3 and 4, by fitting a multivariable Cox regression (Model 3) or using the inverse probability of treatment weighting (Model 4), we observed for all outcomes shifts toward higher HRs compared to the fully crude analysis (Model 1). Most of the findings did not yield statistically significant results, except in Model 1 for the in-hospital death outcome and in Model 3 for the transfer outcome.

In the emulated trial (Model 5) with defining a hypothetical protocol and a reliable 2-day treatment administration period, the resulting HRs were 0.68 (95% CI, 0.46–1.02) for in-hospital death, 1.22 (95% CI, 0.82–1.81) for discharge home, and 1.26 (95% CI, 0.77–2.07) for transfer. The trial emulation analysis allowed to model a hypothetical trial in which the treatment was administered within the first 2 days of hospital admission. This analysis showed that the treatment effect on both discharge home and transfer is toward a beneficial direction, and suggests a reduction in in-hospital death, however none of these results were statistically significant.

## Discussion

This paper provides an overview of the methodological limitations of competing risks, immortal-time bias, and confounding bias when evaluating treatment effectiveness using observational hospital data from COVID-19 patients. This article demonstrates how biases may be mistakenly introduced and discusses the limitations of standard approaches that may lead to biased estimates of treatment effects. Observational studies evaluating treatment effectiveness are often complex, and have the potential for various types of biases. These combinations of biases can result in shifted effects of different magnitudes and directions, making it difficult to accurately estimate treatment effectiveness (14, 19). Our study aims to raise awareness of the common biases and the importance of addressing these limitations. This knowledge is essential for researchers assessing treatment effectiveness, particularly during the emergence or re-emergence of infectious diseases, when investigators face significant time constraints to obtain high-quality evidence of treatment effectiveness when relying on observational data, as was the case during the COVID-19 pandemic.

In our study, we illustrate the competing risk issue using a typical example of observational hospital data. Our results show that the naïve Kaplan-Meier estimator leads to biased cumulative incidence probabilities for the primary event of interest. Censoring discharged patients violated the independent censoring assumption, thus overestimating the probabilities of in-hospital death (47). Various methodologies and analytical techniques are available for analyses in the presence of competing events (48). In our emulated trial study, we used the Aalen-Johansen estimator to account for competing risks (49). This technique determined the proportion of patients who experienced a primary event of interest within the given time, considering the presence of competing events (50). Our previous studies elaborated on implementing competing risk analyses within the target trial emulation framework (51, 52). Another method to account for dependent censoring is to use the inverse probability of censoring weighting, which weights patients by the inverse probability of not yet having the competing event (48, 49). These weights can then be implemented in the Kaplan-Meier estimator (48). In fact, we agree with prior research that the choice of statistical analysis method in the presence of competing events depends on the specific causal research question and the type of event (48).

A competing risk analysis that reports cumulative incidence for heterogeneous outcomes could be particularly beneficial. Acknowledging all clinically important endpoints can provide researchers with a more comprehensive understanding of disease progression and enhance the assessment of therapy-associated benefits and risks. In a target trial emulation study conducted by Urner et al. evaluating the effectiveness of venovenous extracorporeal membrane oxygenation (ECMO) in COVID-19 patients, the study reported results for the primary outcome of in-hospital death and for the competing event of hospital discharge (53). Their study defined discharge alive as a competing event for in-hospital death rather than a censoring event. Such an approach provides a more comprehensive understanding of ECMO’s impact on various clinical outcomes (53).

Previous studies evaluated the impact of immortal-time bias and confounding bias on treatment effect estimates by comparing standard analytical approaches with emulated trials (54, 55). Hoffman et al. (54) reported that immortal time can lead to biased treatment effect estimates. The common “model-first” approaches failed to achieve the randomized controlled trial (RCT) benchmark using the same data source compared to the target trial emulation framework (54). The study conducted by Kuehne et al. evaluated the effectiveness of ovarian cancer treatment in terms of overall survival. The study found that ignoring methodological biases and using crude (univariable) analysis methods led to significant variation in effect measures, with immortal-time bias contributing more substantially to the shifted effects than confounding (55). That study also demonstrated that various methodological biases can significantly shift the treatment effect measure in different directions. Our analysis led to similar conclusions. The magnitude of immortal-time bias can be influenced by factors such as the length of the immortal time period, the proportion of exposed patients, the event rate, and the length of a study’s follow-up (15, 56).

Our study also highlights the impact of baseline confounding bias and the importance of addressing it properly. To prevent confounding bias, it is essential to identify and account for all potential, clinically important confounders, and to apply appropriate statistical methods (27). The evaluation of time-dependent treatments necessitates the inclusion of post-baseline (time-dependent) confounders (54, 57, 58). High-quality, time-dependent data are crucial for drawing causal conclusions from observational data (27, 57). In our analysis, data on time-updated prognostic covariates were not available, which makes our study susceptible to time-dependent confounding bias. This is because treatment administration after baseline often depends on changing prognostic characteristics. To adjust for time-updated covariates, time-dependent clinical characteristics could be incorporated into the weights models (45, 57).

Our examination aligns with the existing literature recommending the target trial emulation framework as a beneficial approach for analyzing real-world data (24, 33, 54). This framework increases transparency in both the design and analysis stages by explicitly defining the research question, outcome, time zero, treatment strategies and assignment, and the analysis plan (24, 33). This approach facilitates the early identification and mitigation of potential biases by applying of design and/or analytical strategies (33). While the target trial emulation framework offers advantages, we acknowledge its methodological complexities and the need to address frequent challenges associated with observational data (24, 59). For more detailed introductions and tutorials on the emulated target trial framework, we refer to the articles by Hernan et al. (33), Fu (24), and Maringe et al. (34).

Our study has several potential limitations. First, it is a demonstrative study that uses a common data example from a single center, restricting the generalizability of our results regarding the magnitude of biases on the treatment effect. Therefore, our findings on the magnitude of each bias cannot be extrapolated to other settings. Second, we developed a simplified version of a hypothetical trial protocol, and additional criteria could be included in real treatment assessment studies. Third, while we accounted for numerous baseline clinical covariates to control for confounding, we admit that unmeasured confounding is probable in our study. Data on time-updated prognostic covariates were not available. Fourth, we reported HRs as a summary measure to facilitate comparisons among the various regression models. Such summary effect measures as risk differences and risk ratios are preferable to hazards and are easier to interpret clinically (47, 60). Lastly, we did not discuss additional limitations of observational studies, such as selection bias, data quality, and missing data issues, all of which can impact the accuracy of their results (4, 61). However, it is important to emphasize that our findings were not interpreted clinically.

## Data availability statement

The datasets presented in this article are not readily available because data are not accessible for public use. Statistical code is available from the corresponding author upon request. Requests to access the code should be directed to martin.wolkewitz@uniklinik-freiburg.de.

## Ethics statement

The study was conducted according to the guidelines of the Declaration of Helsinki, and approved by the Ethics Committee of Bellvitge University Hospital (PR 128/20). The studies were conducted in accordance with the local legislation and institutional requirements. Informed consent was waived after assessment by the Research Ethics Committee.

## Author contributions

OM: Conceptualization, Formal Analysis, Methodology, Software, Visualization, Writing – original draft. DH: Writing – review & editing. HM: Data curation, Funding acquisition, Writing – review & editing. MM: Data curation, Funding acquisition, Writing – review & editing. MAM: Data curation, Funding acquisition, Writing – review & editing. SR: Data curation, Funding acquisition, Writing – review & editing. MR-R: Data curation, Funding acquisition, Investigation, Writing – review & editing. MW: Conceptualization, Formal Analysis, Funding acquisition, Investigation, Methodology, Supervision, Writing – review & editing.
