# Supplementary material for: Methodological biases in observational hospital studies of COVID-19 treatment effectiveness: pitfalls and potential
Source: Front Med (Lausanne). 2024 Mar 21;11:1362192. doi: 10.3389/fmed.2024.1362192 (PMC10991758; doi:10.3389/fmed.2024.1362192)
Supplement: Supplementary file 1 [file Data_Sheet_1.docx]

**Supplementary material 1**. Protocol and components for hypothetical randomized trial and emulated target trial

| **Protocol components** | **Hypothetical target trial** | **Emulated target trial** |
| --- | --- | --- |
| **Clinical study aim** | To estimate the treatment effect on the 45-day risk of in-hospital death, discharge alive, and transfer to another healthcare facility in patients with COVID-19 | The same |
| **Eligibility criteria** | **Inclusion:**  Patients with COVID-19, defined as PaO_2_/FiO_2_ less than 300 mm/Hg at admission and one of the high-risk categories of inflammation:   - CRP: >102 (mg/L) or - LDH: >394 (U/L) or - D-dimer: >1580 (ng/mL) or - ferritin: >1360 (mcg/L) or - total lymphocyte count: <760 (count ×10^6^/L)   **Exclusion:**   - patients less than 18 years - received treatment before baseline or - re-admitted patients: transferred from another hospital | The same, except that patients with dementia diagnosis were not eligible to receive treatment and thus excluded |
| **Treatment strategies** | Two treatment arms: early treatment administration within the first two days of the hospitalization versus never use | The same |
| **Treatment assignment** | Patients random assignment to single dose “X” treatment | - Non-randomized treatment assignment; assumed conditional on demographic and clinical covariates (confounders) - Treatment assignments based on the patient’s clinical status and the decision of physicians within 2-days of hospital admission |
| **Start and end of follow-up** | - Randomization at hospital admission - Follow-up until in-hospital death, discharge home, transfer to another healthcare facility or administrative study end of follow-up (censoring) for patients alive at 45 days | - Start of follow-up at hospital admission; - Observe patients until their strategy is consistent with the protocol; 1) censoring patients at the time of “X” treatment initiation in the control arm; 2) censoring patients in the control arm if treatment “X” has not been administered until day 2; or in-hospital death, discharge home, transfer to another healthcare facility, or censoring at 45 days |
| **Covariates** | Prognostic variables: sex, age, CCI, and high-risk inflammation markers: CRP, LDH, D-dimer, ferritin, and total lymphocyte count | In addition, we included the date of admission categorized according to three waves |
| **Endpoints** | In-hospital death as a primary endpoint and discharge home, transfer to another healthcare facility as competing events | The same |
| **Causal contrast** | Per-protocol effects | Observational analogue of the per-protocol effect |

**Abbreviations:** CCI, Charlson Comorbidity Index; CRP, C-reactive protein; LDH, lactate dehydrogenase

**Supplementary material 2**. Description of the clone-censor-weight approach

Using the target trial emulation framework, the clinical question of interest was to evaluate the effect of “X” treatment on in-hospital mortality in hospitalized patients with COVID-19, considering treatment effects on competing risks. We applied the clone-censor-weight technique to emulate a hypothetical trial from observational data. We cloned our observed data to both arms: the investigated treatment arm, denoted as “X-treated” and the standard of treatment care, denoted as “non-X-treated,” respectively. We also defined a grace period during which patients could initiate treatment two days after hospital admission. We followed each patient's copy until their treatment strategy was inconsistent with the protocol, the primary outcome, or competing events. We censored patients in each treatment arm when they deviated from the protocol. Patients treated after the grace period were classified to the non-X-treated arm. If patients died or were discharged during the first two days, they were assigned to both arms. Otherwise, patient outcomes were only recorded in the arm in which the patient was consistent with the respective arm. We applied the inverse probability of censoring weights to correct for artificial censoring and its introduced selection bias (1).

**Supplementary material 3.** Standardized differences before and after inverse probability censoring weighting

SMD for *M^*^=1* SMD for *M^*^=2*

**
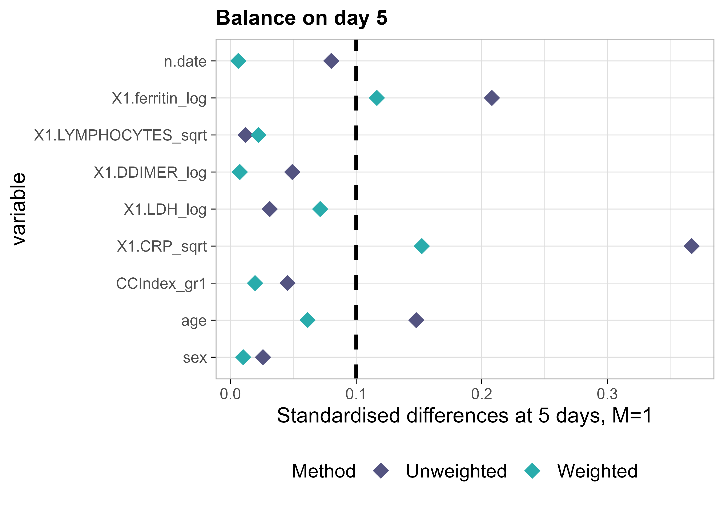
** **
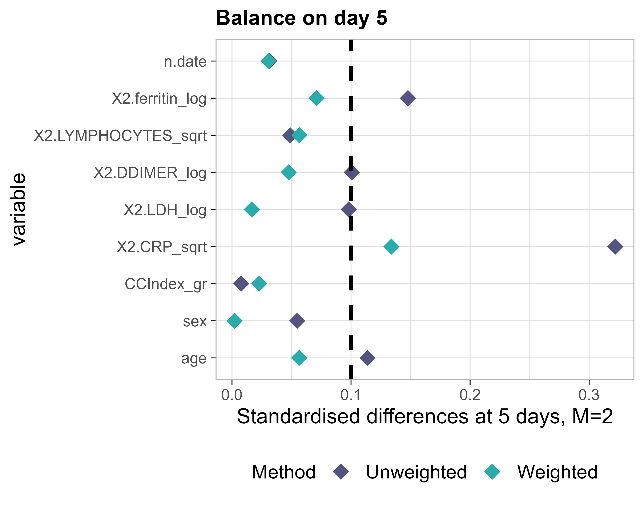
**

SMD for *M^*^=3* SMD for *M^*^=4*

**
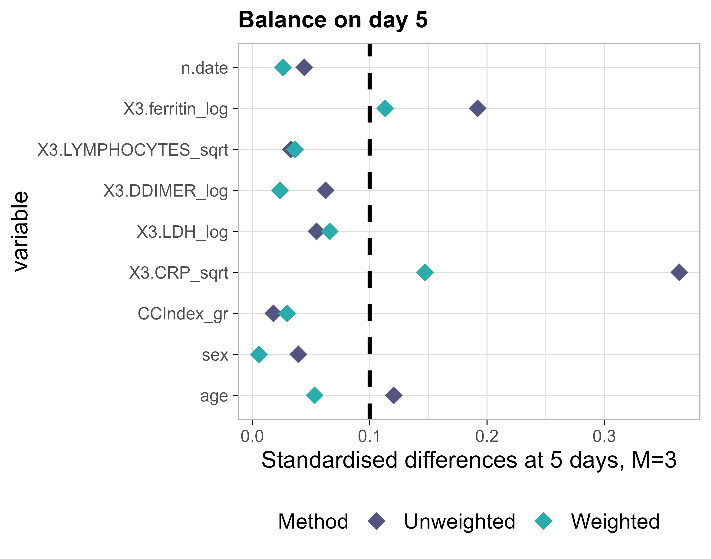
** **
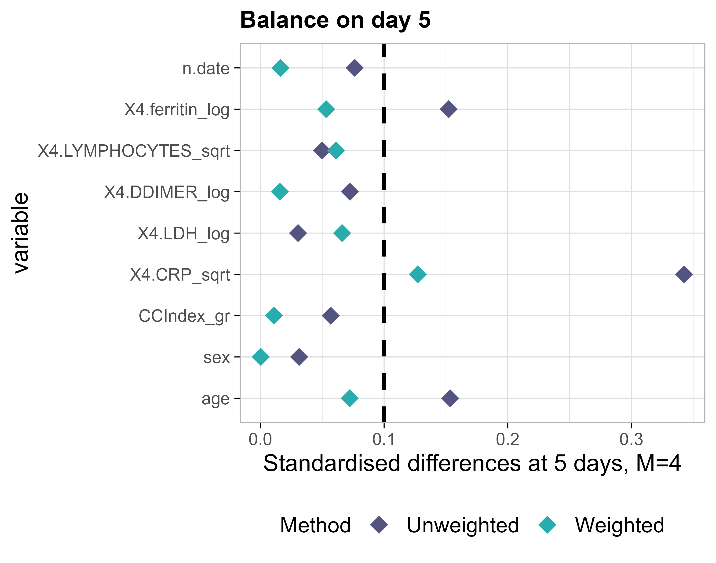
**

SMD for *M^*^=5*

**
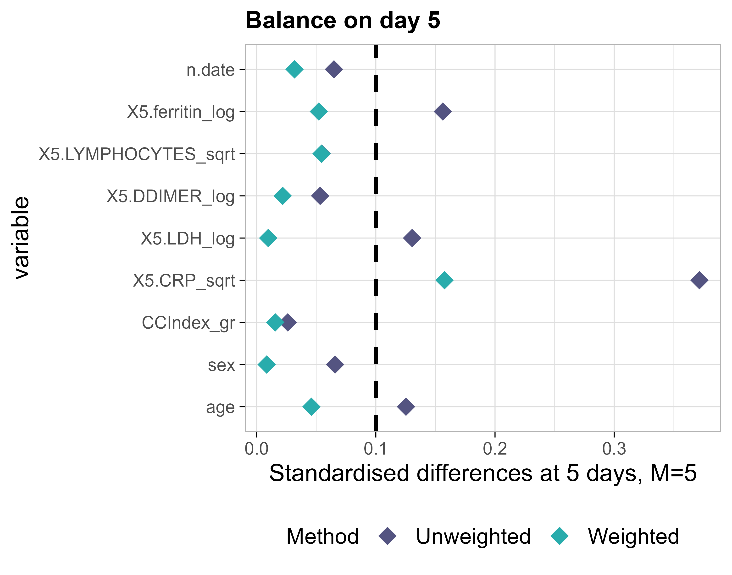
**

**
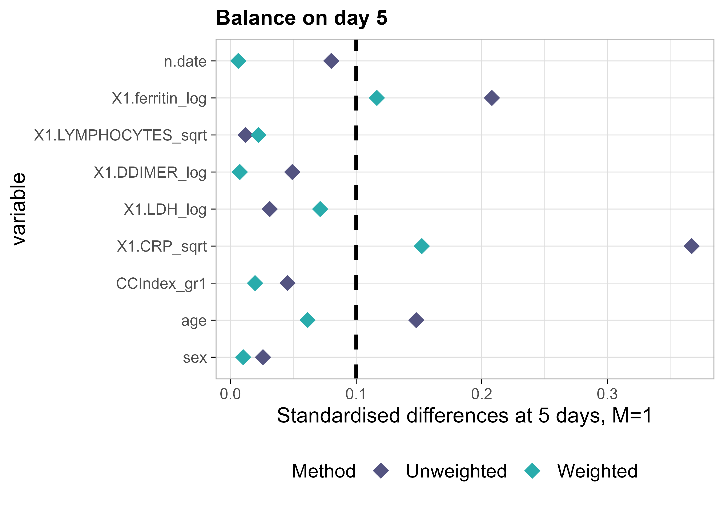
**

**Notes:** ^*^*M – imputed dataset*

**
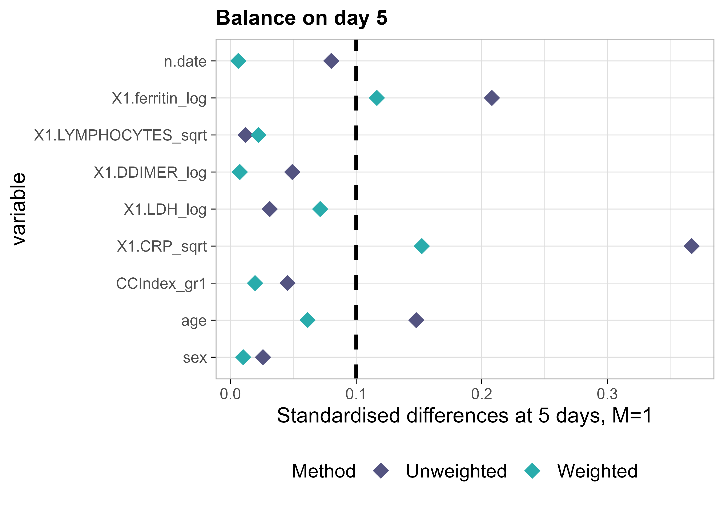
**

**Supplementary material 4.** Missing data imputation

We performed multiple imputations to address missing data for various laboratory inflammatory parameters: C-reactive protein (CRP), lactate dehydrogenase (LDH), ferritin, D-dimer, and lymphocyte count. The distribution of missing values for each baseline covariate was as follows: CRP - 25 (1.2%), LDH - 244 (11.9%), D-dimer - 176 (8.6%), ferritin - 901 (44.0%), and total lymphocyte count - 17 (0.8%). Our imputation strategy was based on multiple imputations, where we created a specific imputation model for each partially observed variable, assuming missing at random (2). However, the missingness of some laboratory parameters was also influenced by physician decisions, indicating that these were missing not at random. For example, ferritin was not routinely collected. The imputation model included all covariates used that we further use in the multivariable or inverse probability treatment/censoring weighting models and also included outcome indicators. We generated five (*M* = 5) imputed datasets, replacing missing values with imputed ones. All the analysis steps were applied to each imputed dataset: selection of eligible patients, inverse probability weighting, survival analysis, and bootstrap variance estimation. Hence, the number of eligible patients slightly varied between datasets as the selection of eligible individuals was conducted after imputation (Table). Once estimates were calculated for each dataset, they then were combined by applying Rubin’s Rules. In Cox regression analyses, we estimated the logarithm of the hazard ratio (HR), and Rubin’s rules were applied to the log(HR) (2,3). The multiple imputation analysis was conducted using the *mice* package in RStudio.

**Table.** Number of eligible COVID-19 patients in each dataset

| **Imputation** | **Number patients** | **Overall number of treated** | **Treated in TTE analysis** | **Outcome^*^** | | | |
| --- | --- | --- | --- | --- | --- | --- | --- |
|  |  |  |  | **In-hospital death** | **Discharge home** | **Transfer** | **Censoring at 45 days** |
| **M=1** | 479 | 142 | 72 | 184 | 139 | 97 | 59 |
| **M=2** | 481 | 144 | 74 | 182 | 141 | 98 | 60 |
| **M=3** | 481 | 144 | 73 | 182 | 140 | 101 | 58 |
| **M=4** | 475 | 143 | 73 | 181 | 139 | 97 | 58 |
| **M=5** | 476 | 142 | 73 | 183 | 138 | 96 | 59 |
| **Average** | 478 | 143 | 73 | 183 | 139 | 98 | 59 |

**Notes:** ^*^ Total number of patients for each outcome included in the final dataset

**Supplementary material 5.** Baseline characteristics for initial cohort and target trial emulation analysis for patients with COVID-19 disease

*Imputed dataset: M=1*

|  | **Initial cohort** | | | **Target trial emulation^*^** | |
| --- | --- | --- | --- | --- | --- |
|  | **overall** | **treated** | **untreated** | **‘X’-treated arm** | **Non-‘X’-treated arm** |
|  | n=479 | n=142 | n=337 | n=72 | n=407 |
| **Age**, median (IQR) | 68 (59-76) | 68 (59-74) | 68  (59-76) | 66  (60-73) | 68  (60-76) |
| **Sex** | | | | | |
| Female, n (%) | 123 (25.7) | 31 (21.8) | 92 (27.3) | 20 (27.8) | 103 (25.3) |
| Male, n (%) | 356 (74.3) | 111 (78.2) | 245 (72.7) | 52 (72.2) | 304 (74.7) |
| **Charlson Comorbidity Index**, >1 (%) | 239 (49.9) | 171 (50.7) | 68 (47.9) | 34 (47.2) | 205 (50.4) |
| **C-reactive protein** (mg/L), median (IQR), | 145.5  (86.5-22.4) | 152.0  (92.0-238.6) | 142.9  (83.2-213.8) | 170.6  (106.4-246.8) | 141.4  (81.9-213.3) |
| **D-dimer** (ng/mL), median (IQR) | 489 (287-1079) | 492  (297-976) | 484  (286-1123) | 554.5  (337-1076) | 455  (285-1077) |
| **Lymphocyte count** ×10^6^/L, median (IQR) | 740  (520-1035) | 740  (543-1040) | 740  (500-1030) | 735 (508-1040) | 455 (285-1077) |
| **Ferritin** (mcg/L), median (IQR) | 1284.0  (631.0-2146.0) | 740.0  (542.5-1040.0) | 1143.0  (560.0-2099.0) | 1504.2 (870.1-2078.6) | 1227.8 (577.8-2147.2) |
| **Lactate dehydrogenase** (U/L), median (IQR) | 430  (331-547) | 423  (332-528) | 431  (329-550) | 420 (363-527) | 430 (324-549) |
| **Wave** | | | | | |
| First, n (%) | 298 (62.2) | 105 (73.9) | 193 (57.3) | 45 (62.5) | 253 (62.2) |
| Second, n (%) | 103 (21.5) | 21 (14.8) | 82 (24.3) | 17 (23.6) | 86 (21.1) |
| Third, n (%) | 78 (16.3) | 16 (11.3) | 62 (18.4) | 10 (13.9) | 68 (16.7) |
| **Outcome** | | | | | |
| In-hospital death, n (%) | 184 (38.4) | 45 (31.7) | 139 (41.2) | 20 (27.8) | 164 (40.3) |
| Discharge home, n (%) | 139 (29.9) | 43 (30.3) | 96 (28.5) | 26 (36.1) | 113 (27.8) |
| Transfer, n (%) | 97 (20.3) | 39 (27.5) | 58 (17.2) | 18 (25.0) | 79 (27.8) |
| Administrative censoring, n (%) | 59 (12.3) | 15 (10.6) | 44 (13.1) | 8 (11.1) | 51 (12.5) |

**Notes:**^*^ characteristics before cloning, censoring, weighting

IQR, Interquartile range

*Imputed dataset: M=2*

|  | **Initial cohort** | | | **Target trial emulation^*^** | |
| --- | --- | --- | --- | --- | --- |
|  | **overall** | **treated** | **untreated** | **‘X’-treated arm** | **Non-‘X’-treated arm** |
|  | n=481 | n=144 | n=337 | n=74 | n=407 |
| **Age**, median (IQR) | 68 (59-76) | 68 (59-75) | 68  (59-76) | 67  (58-73) | 68  (60-76) |
| **Sex** | | | | | |
| Female, n (%) | 122 (25.4) | 32 (22.2) | 90 (26.7) | 21 (28.4) | 101 (24.8) |
| Male, n (%) | 359 (74.6) | 112 (77.8) | 247 (73.3) | 53 (71.6) | 306 (75.2) |
| **Charlson Comorbidity Index**, >1 (%) | 240 (49.9) | 70 (48.6) | 170 (50.4) | 36 (48.6) | 204 (50.1) |
| **C-reactive protein** (mg/L), median (IQR), | 145.5  (86.7-219.8) | 150.6  (91.2-237.9) | 143.90  (83.2-213.8) | 166.7  (102.5-245.3) | 142.0  (81.9-213.3) |
| **D-dimer** (ng/mL), median (IQR) | 462  (289-976) | 481  (285-955) | 455  (291-988) | 555  (336-1050) | 443  (2845-953) |
| **Lymphocyte count** ×10^6^/L, median (IQR) | 740  (520-1030) | 755  (558-1060) | 740  (500-1010) | 755  (513-1055) | 740  (520-1020) |
| **Ferritin** (mcg/L), median (IQR) | 1284.0  (591.3-2070.0) | 1510.4  (974.2-2228.2) | 1075.1  (537.3-1931.6) | 1496.0  (879.1-1955.9) | 1209.0  (573.0-2099.0) |
| **Lactate dehydrogenase** (U/L), median (IQR) | 428  (327-538) | 420  (327-519) | 430  (329-539) | 420  (362-522) | 429  (322-538) |
| **Wave** | | | | | |
| First, n (%) | 298 (62.0) | 105 (72.9) | 193 (57.3) | 45 (60.8) | 253 (62.2) |
| Second, n (%) | 104 (21.6) | 22 (15.3) | 82 (24.3) | 18 (24.3) | 86 (21.1) |
| Third, n (%) | 79 (16.4) | 17 (11.8) | 62 (18.4) | 11 (14.9) | 68 (16.7) |
| **Outcome** | | | | | |
| In-hospital death, n (%) | 182 (37.8) | 40 (27.8) | 136 (40.4) | 21 (28.4) | 161 (39.6) |
| Discharge home, n (%) | 141 (29.3) | 43 (29.9) | 98 (29.1) | 26 (35.1) | 115 (28.3) |
| Transfer, n (%) | 98 (20.4) | 46 (31.9) | 58 (17.2) | 19 (25.7) | 79 (19.4) |
| Administrative censoring, n (%) | 60 (12.5) | 15 (10.4) | 45 (13.4) | 8 (10.8) | 52 (12.8) |

**Notes:**^*^ characteristics before cloning, censoring, weighting

IQR, Interquartile range

*Imputed dataset: M=3*

|  | **Initial cohort** | | | **Target trial emulation^*^** | |
| --- | --- | --- | --- | --- | --- |
|  | **overall** | **treated** | **untreated** | **‘X’-treated arm** | **Non-‘X’-treated arm** |
|  | n=481 | n=144 | n=337 | n=73 | n=408 |
| **Age**, median (IQR) | 68 (59, 76) | 68 (58, 75) | 68 (59, 76) | 67 (58, 73) | 68 (59, 76) |
| **Sex** | | | | | |
| Female, n (%) | 121 (25.2) | 31 (21.5) | 90 (26.7) | 20 (27.4) | 101 (24.8) |
| Male, n (%) | 360 (74.8) | 113 (78.5) | 247 (73.3) | 53 (72.6) | 307 (75.2) |
| **Charlson Comorbidity Index**, >1 (%) | 238 (49.5) | 69 (47.9) | 169 (50.1) | 35 (47.9) | 203 (49.8) |
| **C-reactive protein** (mg/L), median (IQR), | 144.0  (84.0, 219.8) | 150.6  (91.2, 237.9) | 142.0  (81.9, 213.3) | 168.4 (103.0, 246.0) | 140.8 (81.6, 212.5) |
| **D-dimer** (ng/mL), median (IQR) | 489  (291, 1067) | 492  (293, 964) | 489  (291, 1114) | 557  (338, 1073) | 477  (285, 1062) |
| **Lymphocyte count** ×10^6^/L, median (IQR) | 740  (520, 1030) | 750  (558, 1060) | 740  (500, 1010) | 750  (510, 1040) | 740  (520, 1020) |
| **Ferritin** (mcg/L), median (IQR) | 1300.4  (633.1, 2070.0) | 1518.8  (877.1, 2173.6) | 1209.0  (560.0, 2004.5) | 1526.8  (846.1, 2018.5) | 1236.6  (586.2, 2071.9) |
| **Lactate dehydrogenase** (U/L), median (IQR) | 429  (332, 542) | 429  (332, 542) | 429  (332, 542) | 416  (361, 523) | 430  (326, 543) |
| **Wave** | | | | | |
| First, n (%) | 299 (62.2) | 106 (73.6) | 193 (57.3) | 45 (61.6) | 254 (62.3) |
| Second, n (%) | 105 (21.8) | 21 (14.6) | 84 (24.9) | 17 (23.3) | 88 (21.6) |
| Third, n (%) | 77 (16.8) | 17 (11.8) | 60 (17.8) | 11 (15.1) | 66 (16.2) |
| **Outcome** | | | | | |
| In-hospital death, n (%) | 182 (37.8) | 45 (31.3) | 137 (40.7) | 20 (27.4) | 162 (39.7) |
| Discharge home, n (%) | 140 (29.1) | 43 (29.9) | 97 (28.8) | 26 (35.6) | 114 (27.9) |
| Transfer, n (%) | 101 (21.0) | 41 (28.5) | 60 (17.8) | 19 (26.0) | 82 (20.1) |
| Administrative censoring, n (%) | 58 (12.1) | 15 (10.4) | 43 (12.8) | 8 (11.0) | 50 (12.3) |

**Notes:**^*^ characteristics before cloning, censoring, weighting

IQR, Interquartile range

*Imputed dataset: M=4*

|  | **Initial cohort** | | | **Target trial emulation^*^** | |
| --- | --- | --- | --- | --- | --- |
|  | **overall** | **treated** | **untreated** | **‘X’-treated arm** | **Non-‘X’-treated arm** |
|  | n=475 | n=143 | n=332 | n=73 | n=402 |
| **Age**, median (IQR) | 68  (59, 76) | 68  (58, 74) | 68  (59, 76) | 65  (58, 73) | 69  (59, 76) |
| **Sex** | | | | | |
| Female, n (%) | 120 (25.3) | 31 (21.7) | 89 (26.8) | 20 (27.4) | 100 (24.9) |
| Male, n (%) | 355 (74.7) | 112 (78.3) | 243 (73.2) | 53 (72.6) | 302 (75.1) |
| **Charlson Comorbidity Index**, >1 (%) | 236 (49.7) | 68 (47.6) | 168 (50.6) | 34 (46.6) | 202 (50.2) |
| **C-reactive protein** (mg/L), median (IQR), | 146.8  (88.2, 220.4) | 151.1  (92.7, 238.2) | 143.9  (88.0, 213.9) | 168.4  (107.5, 246.0) | 142.4  (85.2, 213.3) |
| **D-dimer** (ng/mL), median (IQR) | 491  (297, 1064) | 494  (290, 999) | 490  (300, 1092) | 557  (338, 1073) | 468  (286, 1052) |
| **Lymphocyte count** ×10^6^/L, median (IQR) | 740  (515, 1020) | 750  (555, 1050) | 735  (500, 1010) | 750  (510, 1040) | 735  (520, 1018) |
| **Ferritin** (mcg/L), median (IQR) | 1295.0  (633.1, 2056.5) | 1481.1  (931.0, 2171.3) | 1481.1  (931.0, 2171.3) | 1481.1  (846.1, 2073.1) | 1239.5  (571.3, 2042.2) |
| **Lactate dehydrogenase** (U/L), median (IQR) | 430  (334, 548) | 416  (326, 521) | 432  (338, 556) | 416  (361, 523) | 430  (333, 551) |
| **Wave** | | | | | |
| First, n (%) | 294 (61.9) | 105 (73.4) | 189 (56.9) | 45 (61.6) | 249 (61.9) |
| Second, n (%) | 104 (21.9) | 22 (15.4) | 82 (24.7) | 18 (24.7) | 86 (21.4) |
| Third, n (%) | 77 (16.2) | 16 (11.2) | 61 (18.4) | 10 (13.7) | 67 (16.7) |
| **Outcome** | | | | | |
| In-hospital death, n (%) | 181 (38.1) | 45 (31.5) | 136 (41.0) | 20 (27.4) | 161 (40.0) |
| Discharge home, n (%) | 139 (29.3) | 43 (30.1) | 96 (28.9) | 26 (35.6) | 113 (28.1) |
| Transfer, n (%) | 97 (20.4) | 40 (28.0) | 57 (17.2) | 19 (26.0) | 78 (19.4) |
| Administrative censoring, n (%) | 58 (12.2) | 15 (10.5) | 43 (13.0) | 8 (11.0) | 50 (12.4) |

**Notes:**^*^ characteristics before cloning, censoring, weighting

IQR, Interquartile range

*Imputed dataset: M=5*

|  | **Initial cohort** | | | **Target trial emulation^*^** | |
| --- | --- | --- | --- | --- | --- |
|  | **overall** | **treated** | **untreated** | **‘X’-treated arm** | **Non-‘X’-treated arm** |
|  | n=476 | n=142 | n=334 | n=73 | n=403 |
| **Age**, median (IQR) | 68  (59, 76) | 68  (59, 74) | 68  (59, 76) | 67  (58, 73) | 68  (59, 76) |
| **Sex** | | | | | |
| Female, n (%) | 121 (25.4) | 32 (22.5) | 89 (26.6) | 21 (28.8) | 100 (24.8) |
| Male, n (%) | 355 (74.6) | 110 (77.5) | 245 (73.4) | 52 (71.2) | 303 (75.2) |
| **Charlson Comorbidity Index**, >1 (%) | 238 (50.0) | 68 (47.9) | 170 (50.9) | 35 (47.9) | 203 (50.4) |
| **C-reactive protein** (mg/L), median (IQR), | 147.4  (88.1, 221.0) | 153.4  (94.9, 242.1) | 144.8  (87.0, 214.2) | 172.7  (107.5, 249.1) | 143.9  (83.6, 213.6) |
| **D-dimer** (ng/mL), median (IQR) | 487  (290, 1062) | 493  (299, 1008) | 483  (287, 1087) | 552  (335, 1073) | 455  (285, 1045) |
| **Lymphocyte count** ×10^6^/L, median (IQR) | 740  (510, 1020) | 750  (535, 1040) | 735  (500, 1010) | 750  (510, 1040) | 740  (510, 1010) |
| **Ferritin** (mcg/L), median (IQR) | 1328.0  (621.8, 1978.1) | 1487.5  (878.5, 2172.2) | 1228.4  (560.5, 1966.5) | 1471.5  (846.1, 1958.2) | 1290.7  (595.8, 1978.9) |
| **Lactate dehydrogenase** (U/L), median (IQR) | 423  (326, 539) | 427  (334, 522) | 422  (325, 542) | 423  (363, 539) | 423  (322, 540) |
| **Wave** | | | | | |
| First, n (%) | 297 (62.4) | 105 (73.9) | 192 (57.5) | 45 (61.6) | 252 (62.5) |
| Second, n (%) | 103 (21.6) | 21 (14.8) | 82 (24.6) | 18 (24.7) | 85 (21.1) |
| Third, n (%) | 76 (16.0) | 16 (11.3) | 60 (18.0) | 10 (13.7) | 66 (16.4) |
| **Outcome** | | | | | |
| In-hospital death, n (%) | 183 (38.4) | 46 (32.4) | 137 (41.0) | 21 (28.8) | 162 (40.2) |
| Discharge home, n (%) | 138 (29.0) | 42 (29.6) | 96 (28.7) | 26 (35.6) | 112 (27.8) |
| Transfer, n (%) | 96 (20.2) | 39 (27.5) | 57 (17.1) | 18 (24.7) | 78 (19.4) |
| Administrative censoring, n (%) | 59 (12.4) | 15 (10.6) | 44 (13.2) | 8 (11.0) | 51 (12.7) |

**Notes:**^*^ characteristics before cloning, censoring, weighting

IQR, Interquartile range

**References**

1. Maringe C, Benitez Majano S, Exarchakou A, Smith M, Rachet B, Belot A, et al. Reflection on modern methods: trial emulation in the presence of immortal-time bias. Assessing the benefit of major surgery for elderly lung cancer patients using observational data. Int J Epidemiol (2020) 49:1719–29. doi:10.1093/ije/dyaa057
2. Leyrat C, Seaman SR, White IR, Douglas I, Smeeth L, Kim J, et al. Propensity score analysis with partially observed covariates: How should multiple imputation be used? Stat Methods Med Res (2019) 28:3–19. doi:10.1177/0962280217713032
3. Hajage D, Combes A, Guervilly C, Lebreton G, Mercat A, Pavot A, et al. Extracorporeal Membrane Oxygenation for Severe Acute Respiratory Distress Syndrome Associated with COVID-19: An Emulated Target Trial Analysis. Am J Respir Crit Care Med (2022) 206:281–94. doi:10.1164/rccm.202111-2495OC
